# Immunosenescence in Testicular Cancer Survivors: Potential Implications of Cancer Therapies and Psychological Distress

**DOI:** 10.3389/fonc.2020.564346

**Published:** 2021-01-15

**Authors:** Silvia De Padova, Milena Urbini, Giuseppe Schepisi, Alessandra Virga, Elena Meggiolaro, Lorena Rossi, Francesco Fabbri, Tatiana Bertelli, Paola Ulivi, Federica Ruffilli, Chiara Casadei, Giorgia Gurioli, Giovanni Rosti, Luigi Grassi, Ugo De Giorgi

**Affiliations:** ^1^ Psycho-Oncology Unit, Istituto Scientifico Romagnolo per lo Studio e la Cura dei Tumori (IRST) IRCCS, Meldola, Italy; ^2^ Biosciences Laboratory, Istituto Scientifico Romagnolo per lo Studio e la Cura dei Tumori (IRST) IRCCS, Meldola, Italy; ^3^ Department of Medical Oncology, Istituto Scientifico Romagnolo per lo Studio e la Cura dei Tumori (IRST) IRCCS, Meldola, Italy; ^4^ Institute of Psychiatry, Department of Biomedical and Specialty Surgical Sciences, University of Ferrara and University Hospital Psychiatry Unit, Integrated Department of Mental Health S. Anna University Hospital and Health Authorities, Ferrara, Italy

**Keywords:** immunosenescence, cancer therapy, chemotherapy, psychological distress, testicular cancer survivors

## Abstract

Testicular cancer (TC) is the most frequent solid tumor diagnosed in young adult males. Although it is a curable tumor, it is frequently associated with considerable short-term and long-term morbidity. Both biological and psychological stress experienced during cancer therapy may be responsible for stimulating molecular processes that induce premature aging and deterioration of immune system (immunosenescence) in TC survivors, leading to an increased susceptibility to infections, cancer, and autoimmune diseases. Immunosenescence is a remodeling of immune cell populations with inversion of the CD4:CD8 ratio, accumulation of highly differentiated memory cells, shrinkage of telomeres, shift of T-cell response to Th2 type, and release of pro-inflammatory signals. TC survivors exposed to chemotherapy show features of immunological aging, including an increase in memory T-cells (CD4+ and CD8+) and high expression of the senescence biomarker p16INK4a in CD3+ lymphocytes. However, the plethora of factors involved in the premature aging of TC survivors make the situation more complex if we also take into account the psychological stress and hormonal changes experienced by patients, as well as the high-dose chemotherapy and hematopoietic stem cell transplantation that some individuals may be required to undergo. The relatively young age and the long life expectancy of TC patients bear witness to the importance of improving quality of life and of alleviating long-term side-effects of cancer treatments. Within this context, the present review takes an in-depth look at the molecular mechanisms of immunosenescence, describing experimental evidence of cancer survivor aging and highlighting the interconnected relationship between the many factors modulating the aging of the immune system of TC survivors.

## Introduction

Testicular cancer (TC) is the most frequent solid tumors in males, accounting for 1–1.5% of all cancers in men. Its incidence is increasing worldwide. Although TC affects relatively young men (between the ages of 20 and 40), it is a curable tumor with a 5-year survival rate of 98% for localized disease (1, 2). Successful management of TC is based on both the correct use of diagnostic tools (including tumor markers) and the selection of the appropriate treatment. In patients with localized disease, surgical treatment may be curative, while in case of recurrent or metastatic disease, platinum-based chemotherapy regimen is the therapy of choice (3, 4). Moreover, patients with progressive disease could receive standard- or high-dose chemotherapy as second line (5–8). In case of high-dose chemotherapy, autologous hematopoietic stem cell transplantation (HSCT) is necessary to restore bone marrow function. The use of high-dose chemotherapy is associated with a high rate of long-term remissions (7–10), however, there are still no prospective studies that have demonstrated an advantage of one chemotherapy approach over another, and both standard-dose and high-dose treatment represent two valid options for patients with relapsed disease (11, 12).

Although the high response rate, TC survivors could develop short- and long-term morbidity. The early onset of age-related diseases and the expression of biological markers indicative of precocious aging of the immune system (known as immunosenescence) have been hypothesized as consequences of the stress caused by the many challenges faced by patients during the course of the disease (including aggressive therapeutic regimens and psychological distress) (13–17).

Given that TC survivors generally have a good life expectancy, their quality of life must be guaranteed. Addressing the long-term effects of TC treatments and the correlated molecular events is thus an important part of patient management. In this review, we focus on the phenomenon of immunosenescence and its mechanisms, contextualized to the types of cellular stress that may affect TC survivors.

## Hallmarks of Immunosenescence

Immunosenescence or immune aging is the functional decline of the adaptive and innate immune systems generally associated with aging (18, 19). During youth, the immune system is quiescent in normal conditions but promptly responds to antigen stimulation. In contrast, the immune system of the elderly is in a state of mild activation and it is not able to adequately response to stimulation. This leads to a chronic pro-inflammatory state that reduces the immune competence of the individual and, from a clinical point of view, it is correlated with a higher rate of morbidity and mortality (19, 20).

**Figure 1:**
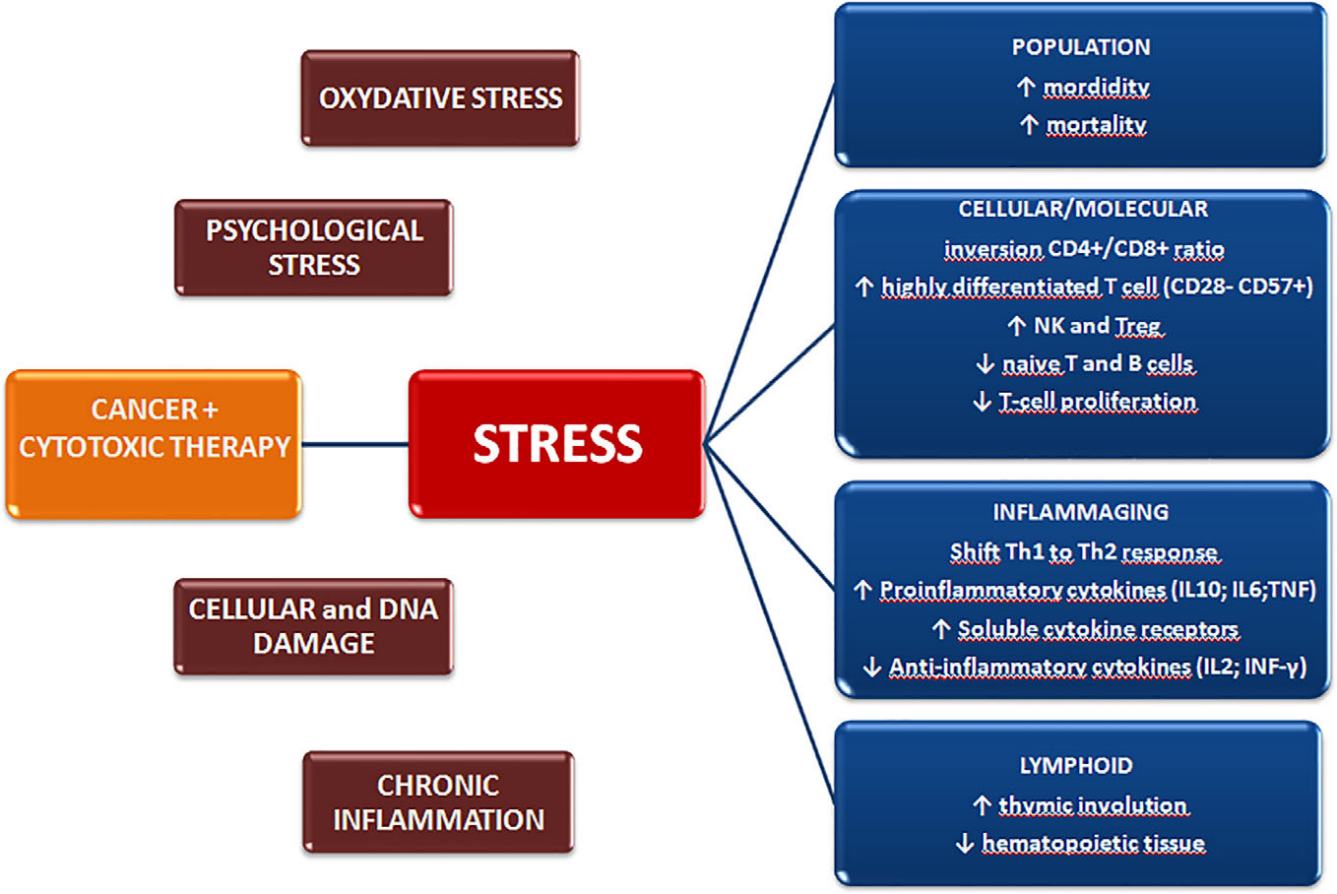
Cancer diagnosis and treatment induce biological and psychological stressors that may promote premature aging of the immune system and exert long-term effects on the quality of life.

Immunosenescence has been widely reported in aged individuals, as a result of the chronic antigen stimulation and cellular stress encountered throughout life (21–23). However, other factors are also associated with the acquisition of precocious senescent features in immune cells, including tissue damage, oxidative stress, cytotoxic therapies, DNA damage, chronic inflammation, and chronic psychological stress (13, 24, 25) (**Figure 1**).

The adaptive response is the immune compartment most widely affected by immunosenescence, with T cell functions undergoing profound and consistent changes during aging (21, 23). A progressive decline in T cell clone expansion in bone marrow and thymus induces a decrease in the number of naïve T lymphocytes. Thymic involution occurs, leading to a progressive decrease in the naïve T-cells exiting the thymus (26, 27). Although the underlying mechanism of this regression is not fully understood, it is believed to be correlated with hormonal changes, oxidative stress, and infections (27, 28). Peripheral expansion is also reduced during aging, mainly due to repetitive stimulation by antigens, which induces a progressive differentiation of T cells. Highly differentiated T cells lose the costimulatory molecule CD28, necessary for TCR signaling, and accumulate features of senescence (*i.e* CD57 and KLRG1), with late-differentiated cells reaching the limit of replication (29) and acquiring markers of senescence (in particular, increase in p16INK4a in peripheral blood CD3+ cells and in SASP phenotype in both T and B cells) (30–33). This results in the filling of immunological space with T-cells with reduced proliferation ability, that secrete pro-inflammatory cytokines (*e.g.* IL10, IL6, and TNF) and that has a reduced T-cell receptor (TCR) repertoire (21, 23, 34). It has also been seen that T-cells may acquire new functions, such as suppressive activity and higher cytotoxic potential (29, 35–38).

Generally, aging brings about an increase in CD8+ T-cells, leading to a decrease in the CD4+/CD8+ ratio as CD4+ T-cells appear to be more resistant to age-related changes. However, an increase in late-memory CD28-negative cells has also been reported in the CD4+ compartment (35, 39, 40). It has been seen that alterations in T-cell functionality may influence polarization of T-helper cells, with a shift in the Th1/Th2-balance to a predominantly Th2 phenotype. In fact, lymphocytes from older individuals produce low levels of the Th1 cytokines IL-2, IFN-γ, and IL-12, while higher levels of the Th2 cytokines, IL-4, IL-5, IL-6, and IL-10 are secreted. This contributes to the altered immune response and to the higher susceptibility to infections seen in elderly people (41).

Several other alterations occurring during aging have also been reported in the B cell compartment, *e.g.* a decrease in naïve B cells and an accumulation of memory CD27 lymphocytes. Moreover, an increase in autoreactive antibody production and a decrease in the BCR repertoire are seen during aging, reflecting the greater susceptibility to autoimmune diseases and the lower responsiveness to infections and vaccinations of the elderly (42).

Immunosenescence is also known to affect innate immunity. Macrophage precursors decrease during aging and show telomere length reduction with decreased secretion of GS-CSF and other cytokines (*e.g.* TNF-alpha and IL-6) (43). Conversely, the number of neutrophils is preserved in the elderly despite the reduction in CD16 Fc expression, indicating an impairment of phagocytosis and of the generation of super-oxide mediated by CD16 Fc receptors (44). Moreover, the cytotoxic activity of natural killer (NK) cells decreases, marked by a reduced interferon secretion, which may affect their ability to eliminate tumors or viral-infected cells (45). Finally, expanded features of immunosuppression have recently been identified during the aging process. Increased numbers of circulating myeloid-derived suppressor cells (MDSCs) and regulatory T-cells (Tregs) have been reported in older mice and humans, further inhibiting the antigen-induced activation of helper and cytotoxic T cells (46, 47).

In brief, immunosenescence is caused by a complex remodeling of all the components of the immune system that may impair its ability to mount an effective defense. Whilst this process is unavoidable during physiological aging, several other pathological conditions may also be capable of impairing immune function.

## Effect of Cancer Treatment Regimens on Immunosenescence

There is increasing evidence that some cancer treatments may induce cellular senescence and aging of the immune system, with implications for the development of secondary medical conditions later in life (13) (**Table 1**). Cytotoxic drugs (including cisplatin, alkylating agents, and anthracyclines) and radiotherapy have been shown to cause cellular senescence in both *in vitro* and *in vivo* models (59–61), with induction of DNA damage, epigenetic alterations and telomere attrition (13, 50, 62). A longitudinal study on breast cancer survivors treated with surgery, radiotherapy or chemotherapy showed that cytokine levels (TNF-alpha and IL-6) were significantly higher in patients than in untreated controls 6 and 18 months after the end of the primary cancer treatment (51). Moreover, expansion of memory T-cell CD28- CD57+ and increment of epigenetic aging were reported after radiotherapy alone or in combination with chemotherapy in breast cancer patients (15, 52), while accumulation of CD57+ T cell was observed in colorectal patients (49). More recently, a study conducted on 60 pediatric-adolescent cancer survivors revealed a higher expression of p16INK4 in CD3+ cells in patients than in the age-matched healthy population 5–6 years after the end of chemotherapy. The increased senescence was associated with the intensity of chemotherapy regimens and the severity of the frailty phenotype of the patient (60).

**Table 1:** Reports of immunosenescence phenotype associated with cancer treatment.

Reference	No. cases	Cancer type	Time after treatment	Group comparison	Immunosenescence evidence
Daniel et al. (16)	44	Pediatric cancer	>10 years	ChT+RT+HSCT *vs* ChT	Accelerated epigenetic aging of T cells and increased production of Th1 cytokines in the irradiated cohort
Lee et al. (48)	21	Pediatric/young adult cancer and other non-malignant diseases	1–3 years	Donor *vs* recipient after allogeneic HSCT	Expansion of senescent T cells (CD28− or CD57+) in recipients, and decrease in ability of cytokines to produce CD4+ cells
Smitherman et al. (49)	69	Pediatric cancer	5–6years	ChT *vs* healthy controls	Increased p16INK4a expression in CD3+ cells of cancer survivors. Positive correlation between p16INK4a expression and patient frailty
Song et al. (50)	2,427	Pediatric cancer	>5 years	ChT, RT *vs* healthy controls	Telomere length was lower in cancer survivors and was associated with chronic health conditions
Alfano et al. (51)	315	Breast cancer	6–18months	ChT, RT *vs* healthy controls	Higher TNF-alpha and IL-6 in treated patients
Onyema et al. (15)	21	Breast cancer	6 months	ChT, RT *vs* healthy controls	Decrease in number of T cells and accumulation of CD28− CD57+ CD8+ T cells in treated patients
Sehl et al. (52)	72	Breast cancer	18 months	Pre- *vs* post-RT or ChT+RT	Increased senescent T cells and epigenetic aging after treatment
Fagnoni et al. (53)	120	Breast cancer	3–5 years	ChT + HSCT *vs* healthy controls	Decrease in number of naive T-cells and higher percentage of CD28− T cells in cancer survivors
Sanoff et al. (54)	33	Breast cancer	3-12 months	Post-ChT *vs* pre-ChT	Higher p16INK4a expression in CD3+ cells after ChT
Wood et al (55).	63	Hematologic malignancies	6 months	Pre- *vs* post-allogeneic or autologous HSCT	Higher p16INK4a expression in CD3+ cells after HSCT, higher p16INK4a expression in allogeneic *vs* autologous HSCT
Bruni et al. (56)	58	Colorectal cancer	ns	ChT-treated *vs* naive patients *vs* healthy controls	Increase in terminally differentiated TEMRA Vδ2pos T cells (CD27−, CD57+) in ChT-treated patients
Laznickova et al. (57)	36	Neuroblastoma	1–4years *vs >*5years	ChT, RT, HSCT *vs* healthy controls	Lower frequency of naıve CD8+ T and higher percentage of CD57+ memory T cells 1–4 years after treatment. At later time point (>5 years), T cell ratio was restored
Bourlon et al. (58)	16	Testicular cancer	3–192 months	ChT *vs* healthy controls	Lower percentage of T-cells, higher number of senescent (CD57+) T-cells and higher p16INK4a expression in CD3+ cells of cancer survivors

ChT, chemotherapy; RT, radiotherapy; HSCT, hematopoietic stem cell transplantation; ns, not specified.

HSCT may also play a role in the induction of immunosenescence in that it induces replicative stress in transplanted stem cells whose job is to reconstitute the immune system (63), causing them to undergo accelerated aging. Consequently, the prevalence of myelodysplasia and secondary malignancies increases after HSCT (64). An increase in senescent features and a reduction in cytokine production have been reported in T-cells of pediatric patients undergoing HSCT (48). Moreover, some studies have analyzed the effect of HSCT combined with total body irradiation or high-dose chemotherapy on the immune system of cancer patients. High-dose chemotherapy combined with autologous stem cell transplantation has been associated with low number of naïve and accumulation of CD28- T-cells, and high expression of senescence markers was detected in CD3+ cells several years after the end of treatment (53, 55). Additionally, chemotherapy in combination with total body irradiation and HSCT has been associated with long-term epigenetic alterations of genes responsible for immune/inflammatory processes and oxidative stress (16). To date, only one paper has described the recovery of the immune system after chemotherapy and HSCT (57). Lázničková et al. reported that, although pediatric patients with neuroblastoma showed features of immunosenescence at early time-points (1–4 years) after treatment, they also began to show signs of immune reconstitution 5 or more years after diagnosis (57). However, the possibility of premature aging of the immune system at later time-points cannot be excluded and, as already stated, recovery has not been reported in survivors of other cancer types (16, 50, 49).

Only one study has been conducted to date on the long-term effects of chemotherapy on the immune system of TC survivors (58). Bourlon et al. compared TC patients undergoing chemotherapy (≥3 cycles of PEB) with a cohort of healthy age-matched men, reporting that cancer patients had significantly lower levels of T cells and CD4+ cells in total lymphocytes than the control group. Increase of memory cells number was detected in both CD4+ and CD8+ compartments, whereas memory B cells unexpectedly decreased. Moreover, CD3+ cells expressed higher levels of p16INK4a, suggesting the induction of a senescent phenotype (58). Even if few cases were analyzed, this is the first study to identify an aged immune system phenotype in TC survivors. Additionally, a correlation between a higher systemic inflammation index and poor prognosis was reported in TC patients, especially in relation to PDL1 expression (65). However, further studies are needed to address the role of other factors specific to a TC context (*e.g.* residual serum platinum levels, psychological stress, and different therapeutic schedules) in the aging phenotype.

## Psychological Stress and Induction of Immunosenescence

The majority of TC survivors report good physical functioning and a relatively high health-related quality of life (66). However, as TC is acknowledged as both a highly distressing and potentially traumatic life event, it may override an individual’s perceived ability to cope with the disease, eliciting emotional, behavioral, and physiological reactions that generate conditions of acute and chronic stress (67–69). The typically young age of patients at diagnosis and the existential challenge of a life-threatening disease and physical complications can interfere with psychological well-being both during and after treatment, inducing psychological distress and morbidity (65). The levels of psychological distress in TC survivors are higher than in noncancerous reference populations, with a more prevalent and severer anxiety (between 9 and 27%) and a greater fear of cancer (70–74). In most studies, depression in TC survivors appeared no more prevalent than in the general population although an Australian study did find higher rates of, and more severe, depression in TC survivors than population norms (73). Cancer-related stress symptomatology such as post-traumatic stress disorder (PTSD) is among the long‐term psychological effects of TC. This disorder includes intrusive ideation, hyper activation, re-experimentation, and avoidance. Dahl et al. observed PTSD in 10.9% of long-term TC survivors at an average of 11 years after diagnosis (75). Psychological stress is known to modulate the immune response and can partially suppress certain aspects of immune function (76, 77). Psychological distress and morbidity in both cancer survivors and the general population can lead to negative changes in bio-behavioral responses (78), inducing detrimental effects on immune health and function and immunological aging (79, 80). In particular, chronic stress, emotional distress, and social difficulties are associated with increased sympathetic nervous system signaling, hypothalamic pituitary adrenal axis dysregulation, inflammation, and decreased cellular immunity. These alterations in physiological adaptation systems are important risk factors for the development and progression of a wide range of physical and mental disorders (14, 79, 81–83).

In a meta-analysis, Segerstrom and Miller found that psychological strain or stress modifies the capacity and regulation of the immune response system and that the consequences of the stressful event vary on the basis of temporal parameters (acute *vs.* chronic) and the type of event (trauma *vs.* loss) (84). Stressful life events and associated negative emotions such as anxiety and depression affect the immune response by altering the sensitive interaction between the central nervous and hormonal systems and the immune system itself (85–88). Prolonged psychological stress appears to be correlated with cellular aging, inducing characteristic senescence features such as increased oxidative stress, reduced telomere length, chronic exposure to glucocorticoids, decreased thymus, changes in cell trafficking, decreased cell-mediated immune response, steroid resistance, and chronic low-grade inflammation (14, 89, 90).

Another important aspect of the relationship between cancer-derived psychological distress and immune system alterations is the fact that exposure to a traumatic event may increase the risk of psychiatric disorders and psychopathological suffering. It is believed that inflammation, one of the hallmarks of immunosenescence, contributes to this (91–93).

Recently, some studies have shown a clear association between PTSD and accelerated cellular senescence, as indicated by decreased telomere length, increased levels of inflammatory cytokines, enhanced T cell responses, a lower frequency of naïve CD8+ T-cells, and a rise in central memory and effector CD8+ T cells (94–96). In addition, symptoms of PTSD have been shown to more frequently associated with an aged immune phenotype characterized by a higher effector memory to naïve CD4+ and CD8+ T cell ratio (96–98).

The relationship between the psychological distress of TC survivors and changes in the immune system has not been widely investigated. However, given that cancer is undoubtedly one of the most stressful events in a person’s life, placing demands on psychological adaptation during treatment and also throughout cancer survivorship (99), studies focusing on the correlation between psychological distress and immunosenescence in TC survivors are warranted.

## Stress-Induced Immunosenescence in Testicular Cancer Survivors

TC survivors have an increased risk to suffer of late adverse events, including hypogonadism, infertility, metabolic syndromes, neurotoxicity, lower cognitive functions, reduced renal function, heart disease, and secondary cancers (100).

Although cisplatin-based chemotherapy is highly effective against TC, its long-term negative effects on healthy tissue have also been reported. In fact, whilst plasma platinum levels show a rapid decline from 1 to 3 years after chemotherapy, platinum has also been detected in the blood up to 20 years after treatment. In TC survivors, platinum concentrations correlated with the cisplatin dose used during therapy have been associated with neurotoxicity and other long-term adverse consequences (101–103). Within this context, it is important to underline the importance of the effects of treatment on a physical level (104), including erectile dysfunction (105), reduction in sexual activity (106), and loss of desire (107). In particular, a correlation between previous chemoradiotherapy and radiotherapy and the risk of erectile dysfunction was recently confirmed by a Danish study on a cohort of 2,260 TC survivors (108). As chemotherapy has a greater impact on Leydig cells than surgery or radiotherapy, TC survivors have a higher risk of hypogonadism (with increasing serum luteinizing hormone concentrations and decreasing serum testosterone levels) (109). A Norwegian study identified four risk factors for the development of hypogonadism, *i.e.* orchiectomy, testicular dysgenesis syndrome, treatment after orchiectomy, and aging (110). This risk was higher in the cohort undergoing chemotherapy than in other cohorts, with luteinizing hormone (LH) levels remaining very high in the former at 20 years. Moreover, the alteration in testosterone and LH levels accelerated over time, leading to the hypothesis of a reduced reserve capacity that worsens as time passes (110).

Hypogonadism also increases the risk of developing metabolic syndrome, including cardiovascular complications and diabetes, which appears to be greater after combination therapy (chemo-radiotherapy) (111, 112). Research is currently underway to evaluate the impact of testosterone administration in long-term TC survivors, but results are still somewhat discordant (113–116).

Moreover, studies focusing on the risk of developing diabetes have reported a potential correlation between low testosterone levels and changes in the immune system. In fact, lower testosterone levels are associated with central adiposity and insulin resistance (117), both of which are frequently involved in inflammatory and oxidative processes and endothelial dysfunction. Liao et al. demonstrated that testosterone levels inhibit inflammation mechanisms (including the TNF-pathway), leading to a modulation of endothelial cell function and promoting wound-healing and angiogenesis. A fundamental role is played by the androgen receptor in this process (118). However, its relationship with metabolic syndrome is unclear (119, 120). Inflammatory states and stress may also be important conditions that lead to cytokine secretion (in particular, IL-6 and CRP), with a subsequent impact on aging. Maggio et al. hypothesized a correlation between low testosterone levels and increased secretion of pro-inflammatory cytokines in the genesis and maintenance of chronic diseases (121).

Several studies have been carried out to evaluate the correlation between treatment-related immunosenescence and the risk of a second cancer in TC survivors, some confirming a higher risk in patients undergoing chemotherapy and radiotherapy than in those treated with surgery alone. Second tumors have mainly been found in organs anatomically near pelvis (122–124) probably related to their closeness to radiation fields or to platinum residue in urothelial tissue. Conversely, cases of secondary leukemia may be related to platinum-dependent alterations in the bone marrow (125). Together with accumulated psychological stress, such factors could influence the aging of the immune system in TC survivors.

## Conclusions and Perspectives

Cancer therapies are life-saving for countless patients but are also associated with side-effects (some long-term) on aging and on the immune system that may be responsible for multiple morbidities (13). Whilst platinum-based therapies are extremely efficacy for recurrent or metastatic TC, platinum is still detectable in the plasma of patients up to 20 years after treatment and it could have deleterious consequence on the organism (101–103). Moreover, also the psychological distress caused by challenges faced during the course of the disease are able to induce the onset of an early-aging phenotype and a decline of the immune system (14). TC survivors struggle with high rates of anxiety and PTSD (73–75) and with the effect of adverse events such as hypogonadism, metabolic disorder, and second malignancies (100). Thus, a better understanding of immunosenescence and of its molecular mechanisms is needed to improve therapeutic options and mitigate their late-effects on cancer patients. Features of senescence involving the adaptive immune response have been reported in TC survivors treated with three or more cycles of chemotherapy (58), however, the overall picture of the long-term effect of current TC management on the immune system is still incomplete. Other studies are needed to address how psychological stress caused by the diagnosis and treatment of a life-threatening disease could affect the immune system. Numerous other molecular aspects accelerating the aging process could also be studied in this cancer setting. A better comprehension of immunosenescence in the general population and of its causes and effects in cancer patients is desirable, hoping to shed the light to some events that could be improved to ameliorate the management of TC survivors. Further research is warranted to identify factors associated with immune aging, late complications and stress in TC survivors, and screening programs should be inserted into follow-up programs.

## Author Contributions

SP, UG, and MU conceptualized and designed the study. MU, SP, and GS drafted the manuscript. GR and LG supervised the project. PU, FF, AV, and GG contributed to the writing and critical discussion of molecular aspects. EM, TB, FR, and LG contributed to the writing and critical discussion of psychological aspects. UG, LR, CC, and GR contributed to the writing and critical discussion of TC medical aspects. All authors contributed to the article and approved the submitted version.

## Conflict of Interest

UG has received advisory board or consultant fees from Merck Sharp & Dohme, Bristol Myers Squibb, Janssen, Astellas, Sanofi, Bayer, Pfizer, Ipsen, Novartis, Pharmamar, and an institutional research grant from Astrazeneca, Sanofi, and Roche.

The remaining authors declare that the research was conducted in the absence of any commercial or financial relationships that could be construed as a potential conflict of interest.

## References

[1] Chia VM Quraishi SM Devesa SS Purdue MP Cook MB McGlynn KA International trends in the incidence of TC, 1973–2002. Cancer Epidemiol Biomarkers Prev (2010) 19:1151–9. 10.1158/1055-9965.EPI-10-0031 PMC2867073 20447912

[2] Stang A Jansen L Trabert B Rusner C Eberle A Katalinic A Survival after a diagnosis of testicular germ cell cancers in Germany and the United States, 2002-2006: a high resolution study by histology and age. Cancer Epidemiol (2013) 37(4):492–7. 10.1016/j.canep.2013.03.017 PMC4024392 23623488

[3] Chovanec M Hanna N Cary KC Einhorn L Albany C Management of stage I testicular germ cell tumours. Nat Rev Urol (2016) 13(11):663–73. 10.1038/nrurol.2016.164 27618772

[4] Albers P Albrecht W Algaba F Bokemeyer C Cohn-Cedermark G Fizazi K Guidelines on testicular cancer: 2015 update. Eur Urol (2015) 68:1054–68. 10.1016/j.eururo.2015.07.044 26297604

[5] Fizazi K Gravis G Flechon A Geoffrois L Chevreau C Laguerre B Combining gemcitabine, cisplatin, and ifosfamide (GIP) is active in patients with relapsed metastatic germ-cell tumors (GCT): a prospective multicenter GETUG phase II trial. Ann Oncol (2014) 25(5):987–91. 10.1093/annonc/mdu099 24595454

[6] De Giorgi U Rosti G Aieta M Testore F Burattini L Fornarini G Phase II study of oxaliplatin and gemcitabine salvage chemotherapy in patients with cisplatin-refractory nonseminomatous germ cell tumor. Eur Urol (2006) 50(5):1032–8. 10.1016/j.eururo.2006.05.011 16757095

[7] De Giorgi U Demirer T Wandt H Taverna C Siegert W Bornhauser M Solid Tumor Working Party of the European Group for Blood and Marrow Transplantation. Second-line high-dose chemotherapy in patients with mediastinal and retroperitoneal primary non-seminomatous germ cell tumors: the EBMT experience. Ann Oncol (2005) 16(1):146–51. 10.1093/annonc/mdi017 15598952

[8] Lorch A Neubauer A Hackenthal M Dieing A Hartmann JT Rick O High-dose chemotherapy (HDCT) as second-salvage treatment in patients with multiple relapsed or refractory germ-cell tumors. Ann Oncol (2010) 21(4):820–5. 10.1093/annonc/mdp366 19822531

[9] De Giorgi U Rosti G Slavin S Yaniv I Harousseau JL Ladenstein R European Group for Blood and Marrow Transplantation Solid Tumours and Paediatric Disease Working Parties. Salvage high-dose chemotherapy for children with extragonadal germ-cell tumours. Br J Cancer (2005) 93(4):412–7. 10.1038/sj.bjc.6602724 PMC2361583 16106248

[10] Simonelli M Rosti G Banna GL Pedrazzoli P Italian Germ cell cancer Group (IGG) Gruppo Italiano Trapianto Midollo Osseo Intensified chemotherapy with stem-cell rescue in germ-cell tumors. Ann Oncol (2012) 23(4):815–22. 10.1093/annonc/mdr403 21948814

[11] Feldman DR1 Bosl GJ Sheinfeld J Motzer RJ Medical treatment of advanced testicular cancer. JAMA (2008) 299(6):672–84. 10.1001/jama.299.6.672 18270356

[12] Fizazi K Oldenburg J Dunant A Chen I Salvioni R Hartmann JT Assessing prognosis and optimizing treatment in patients with postchemotherapy viable nonseminomatous germ-cell tumors (NSGCT): results of the sCR2 international study. Ann Oncol (2008) 19(2):259–64. 10.1093/annonc/mdm472 18042838

[13] Cupit-Link MC Kirkland JL Ness KK Armstrong GT Tchkonia T LeBrasseur NK Biology of premature ageing in survivors of cancer. ESMO Open (2017) 2:e000250. 10.1136/esmoopen-2017-000250 29326844 PMC5757468

[14] Bauer ME Jeckel CM Luz C The Role of Stress Factors during Aging of the Immune System. Ann N Y Acad Sci (2009) 1153:139–52. 10.1111/j.1749-6632.2008.03966.x 19236337

[15] Onyema OO Decoster L Njemini R Forti LN Bautmans I De Waele M Chemotherapy-induced changes and immunosenescence of CD8+ T-cells in patients with breast cancer. Anticancer Res (2015) 35(3):1481–9. 25750301

[16] Daniel S Nylander V Ingerslev LR Zhong L Fabre O Clifford B T cell epigenetic remodeling and accelerated epigenetic aging are linked to long-term immune alterations in childhood cancer survivors. Clin Epigenetics (2018) 10(1):138. 10.1186/s13148-018-0561-5 30400990 PMC6219017

[17] Antoni MH Dhabhar FS The impact of psychosocial stress and stress management on immune responses in patients with cancer. Cancer (2019) 125(9):1417–31. 10.1002/cncr.31943 PMC6467795 30768779

[18] Solana R Pawelec G Molecular and cellular basis of immunosenescence. Mech Ageing Dev (1998) 102(2-3):115–29. 10.1016/S0047-6374(98)00029-3 9720646

[19] Fülöp T Dupuis G Witkowski JM Larbi A The Role of Immunosenescence in the Development of Age-Related Diseases. Rev Invest Clin (2016) 68(2):84–91. 27103044

[20] Shaw AC Goldstein DR Montgomery RR Age-dependent dysregulation of innate immunity. Nat Rev Immunol (2013) 13(12):875–87. 10.1038/nri3547 PMC4096436 24157572

[21] Ventura MT Casciaro M Gangemi S Buquicchio R Immunosenescence in aging: between immune cells depletion and cytokines up-regulation. Clin Mol Allergy (2017) 14;15:21. 10.1186/s12948-017-0077-0 PMC5731094 29259496

[22] Franceschi C Bonafè M Valensin S Olivieri F De Luca M Ottaviani E Inflamm-aging. An evolutionary perspective on immunosenescence. Ann N Y Acad Sci (2000) 908:244–54. 10.1111/j.1749-6632.2000.tb06651.x 10911963

[23] Fulop T Larbi A Dupuis G Le Page A Frost EH Cohen AA Immunosenescence and Inflamm-Aging As Two Sides of the Same Coin: Friends or Foes? Front Immunol (2018) 8:1960. 10.3389/fimmu.2017.01960 29375577 PMC5767595

[24] Coluzzi E Leone S Sgura A Oxidative Stress Induces Telomere Dysfunction and Senescence by Replication Fork Arrest. Cells (2019) 8(1):19. 10.3390/cells8010019 PMC6356380 30609792

[25] Schutte NS Malouff JM The Relationship Between Perceived Stress and Telomere Length: A Meta-analysis. Stress Health (2016) 32(4):313–9. 10.1002/smi.2607 25393133

[26] Douek DC McFarland RD Keiser PH Gage EA Massey JM Haynes BF Changes in thymic function with age and during the treatment of HIV infection. Nature (1998) 396(6712):690–5. 10.1038/25374 9872319

[27] Moro-García MA Alonso-Arias R López-Larrea C Molecular mechanisms involved in the aging of the T-cell immune response. Curr Genomics (2012) 13(8):589–602. 10.2174/138920212803759749 23730199 PMC3492799

[28] Barbouti A Vasileiou PVS Evangelou K Vlasis KG Papoudou-Bai A Gorgoulis VG Implications of Oxidative Stress and Cellular Senescence in Age-Related Thymus Involution. Oxd Med Cell Longev (2020) 2020:7986071. 10.1155/2020/7986071 PMC7025075 32089780

[29] Plunkett FJ Franzese O Finney HM Fletcher JM Belaramani LL Salmon M The loss of telomerase activity in highly differentiated CD8+CD28–CD27– T cells is associated with decreased Akt (Ser473) phosphorylation. J Immunol (2007) 178:7710–9. 10.4049/jimmunol.178.12.7710 17548608

[30] Campisi J Aging, cellular senescence, and cancer. Annu Rev Physiol (2013) 75:685–705. 10.1146/annurev-physiol-030212-183653 23140366 PMC4166529

[31] Liu Y Sanoff HK Cho H Burd CE Torrice C Ibrahim JG Expression of p16INK4a in peripheral blood T-cells is a biomarker of human aging. Aging Cell (2009) 8:439–48. 10.1111/j.1474-9726.2009.00489.x PMC2752333 19485966

[32] Callender LA Carroll EC Beal RWJ Chambers ES Nourshargh S Akbar AN Human CD8+ EMRA T cells display a senescence-associated secretory phenotype regulated by p38 MAPK. Aging Cell (2018) 17(1):e12675. 10.1111/acel.12675 PMC5770853 29024417

[33] Frasca D Diaz A Romero M Blomberg BB Human Peripheral Late/Exhausted Memory B Cells Express a Senescent-Associated Secretory Phenotype and Preferentially Utilize Metabolic Signaling Pathways. Exp Gerontol (2017) 87(Pt A):113–20. 10.1016/j.exger.2016.12.001 27931848

[34] Qi Q Liu Y Cheng Y Glanville J Zhang D Lee JY Diversity and clonal selection in the human T-cell repertoire. Proc Natl Acad Sci U S A (2014) 111(36):13139–44. 10.1073/pnas.1409155111 PMC4246948 25157137

[35] Strioga M Pasukoniene V Characiejus D CD8+ CD28- and CD8+ CD57+ T cells and their role in health and disease. Immunology (2011) 134(1):17–32. 10.1111/j.1365-2567.2011.03470.x 21711350 PMC3173691

[36] Vallejo AN CD28 extinction in human T cells: altered functions and the program of T-cell senescence. Immunol Rev (2005) 205:158–69. 10.1111/j.0105-2896.2005.00256.x 15882352

[37] Henson SM Franzese O Macaulay R Libri V Azevedo RI Kiani-Alikhan S KLRG1 signaling induces defective Akt (ser473) phosphorylation and proliferative dysfunction of highly differentiated CD8+ T cells. Blood (2009) 113(26):6619–28. 10.1182/blood-2009-01-199588 19406987

[38] Pangrazzi L Reidla J Carmona Arana JA Naismith E Miggitsch C Meryk A CD28 and CD57 define four populations with distinct phenotypic properties within human CD8(+) T cells. Eur J Immunol (2020) 50(3):363–79. 10.1002/eji.201948362 PMC7079235 31755098

[39] Weinberger B Lazuardi L Weiskirchner I Keller M Neuner C Fischer KH Healthy aging and latent infection with CMV lead to distinct changes in CD8+ and CD4+ T-cell subsets in the elderly. Hum Immunol (2007) 68(2):86–90. 10.1016/j.humimm.2006.10.019 17321897

[40] Weng NP Akbar AN Goronzy J CD28(–) T cells: their role in the age-associated decline of immune function. Trends Immunol (2009) 30:306–12. 10.1016/j.it.2009.03.013 PMC2801888 19540809

[41] Uciechowski P Kahmann L Plumakers B Malavolta M Mocchegiani E Dedoussis G TH1 and TH2 cell polarization increases with aging and is modulated by zinc supplementation. Exp Gerontol (2008) 43:493–8. 10.1016/j.exger.2007.11.006 18166287

[42] Barber DL Wherry EJ Masopust D Zhu B Allison JP Sharpe AH Restoring function in exhausted CD8 T cells during chronic viral infection. Nature (2006) 439(7077):682–7. 10.1038/nature04444 16382236

[43] Della Bella S Bierti L Presicce P Arienti R Valenti M Saresella M Peripheral blood dendritic cells and monocytes are differently regulated in the elderly. Clin Immunol (2007) 122:220–8. 10.1016/j.clim.2006.09.012 17101294

[44] Butcher SK Chahal H Nayak L Sinclair A Henriquez NV Sapey E Senescence in innate immune responses: reduced neutrophil phagocytic capacity and CD16 expression in elderly humans. J Leukoc Biol (2001) 70:881–6. 11739550

[45] Solana R Campos C Pera A Tarazona R Shaping of NK cell subsets by aging. Curr Opin Immunol (2014) 29:56–61. 10.1016/j.coi.2014.04.002 24792889

[46] Garg SK Delaney C Toubai T Ghosh A Reddy P Banerjee R Aging is associated with increased regulatory T-cell function. Aging Cell (2014) 3):441–8. 10.1111/acel.12191 PMC4032602 24325345

[47] Verschoor CP Johnstone J Millar J Dorrington MG Habibagahi M Lelic A Blood CD33(+)HLA-DR(-) myeloid-derived suppressor cells are increased with age and a history of cancer. J Leukoc Biol (2013) 93(4):633–7. 10.1189/jlb.0912461 PMC3701116 23341539

[48] Lee GH Hong KT Choi JY Shin HY Lee WW Kang HJ Immunosenescent characteristics of T cells in young patients following haploidentical haematopoietic stem cell transplantation from parental donors. Clin Transl Immunol (2020) 9(4):e1124. 10.1002/cti2.1124 PMC7142179 32280463

[49] Smitherman BA Wood WA Mitin N Ayer Miller VL Deal AM Davis IJ Accelerated aging among childhood, adolescent, and young adult cancer survivors is evidenced by increased expression of p16INK4a and frailty. Cancer (2020) 126(22):4975–83. 10.1002/cncr.33112 PMC7607511 32830315

[50] Song N Li Z Qin N Howell CR Wilson CL Easton J Shortened Leukocyte Telomere Length Associates with an Increased Prevalence of Chronic Health Conditions among Survivors of Childhood Cancer: A Report from the St. Jude Lifetime Cohort. Clin Cancer Res (2020) 26(10):2362–71. 10.1158/1078-0432.CCR-19-2503 PMC7231652 31969337

[51] Alfano CM Peng J Andridge RR Lindgren ME Povoski SP Lipari AM Inflammatory cytokines and comorbidity development in breast cancer survivors versus noncancer controls: evidence for accelerated aging? J Clin Oncol (2016) 34:149–56. 10.1200/JCO.2016.67.1883 PMC5455675 27893337

[52] Sehl ME Carroll JE Horvath S Bower JE The acute effects of adjuvant radiation and chemotherapy on peripheral blood epigenetic age in early stage breast cancer patients. NPJ Breast Cancer (2020) 6:23. 10.1038/s41523-020-0161-3. eCollection 2020. 32566744 PMC7293278

[53] Fagnoni F Lozza L Zambelli A Ponchio L Gibelli N T-cell dynamics after high-dose chemotherapy in adults: elucidation of the elusive CD8+ subset reveals multiple homeostatic T-cell compartments with distinct implications for immune competence. Immunology (2002) 106(1):27–37. 10.1046/j.1365-2567.2002.01400.x 11972629 PMC1782702

[54] Sanoff HK Deal AM Krishnamurthy J Torrice C Dillon P Sorrentino J Effect of cytotoxic chemotherapy on markers of molecular age in patients with breast cancer. J Natl Cancer Inst (2014) 106(4):dju057. 10.1093/jnci/dju057 24681605 PMC3982894

[55] Wood WA Krishnamurthy J Mitin N Torrice C Parker JS Snavely AC Chemotherapy and Stem Cell Transplantation Increase p16INK4a Expression, a Biomarker of T-cell Aging. EBioMedicine (2016) 11:227–38. 10.1016/j.ebiom.2016.08.029 PMC5049997 27591832

[56] Bruni E Cazzetta V Donadon M Cimino M Torzilli G Spata G Chemotherapy accelerates immune-senescence and functional impairments of Vδ2pos T cells in elderly patients affected by liver metastatic colorectal cancer. J Immunother Cancer (2019) 7(1):347. 10.1186/s40425-019-0825-4 31829255 PMC6907143

[57] Lázničková P Kepák T Hortová-Kohoutková M Horváth L Sheardová K Marciniak R Childhood survivors of high-risk neuroblastoma show signs of immune recovery and not immunosenescence. Eur J Immunol (2020) 50(12):2092–4. 10.1002/eji.202048541 PMC7754117 32744364

[58] Bourlon MT Velazquez HE Hinojosa J Orozco L Rios-Corzo R Lima G Immunosenescence profile and expression of the aging biomarker (p16INK4a) in testicular cancer survivors treated with chemotherapy. BMC Cancer (2020) 20(1):882. 10.1186/s12885-020-07383-2 32928147 PMC7491179

[59] Roninson IB Tumor cell senescence in cancer treatment. Cancer Res (2003) 63(11):2705–15. 12782571

[60] Piegari E De Angelis A Cappetta D Russo R Esposito G Costantino S Doxorubicin induces senescence and impairs function of human cardiac progenitor cells. Basic Res Cardiol (2013) 108(2):334. 10.1007/s00395-013-0334-4 23411815

[61] Mikuła-Pietrasik J Niklas A Uruski P Tykarski A Książek K Mechanisms and significance of therapy-induced and spontaneous senescence of cancer cells. Cell Mol Life Sci (2020) 77(2):213–29. 10.1007/s00018-019-03261-8 PMC6970957 31414165

[62] Townsley DM Dumitriu B Liu D Biancotto A Weinstein B Chen C Danazol Treatment for Telomere Diseases. N Engl J Med (2016) 374(20):1922–31. 10.1056/NEJMoa1515319 PMC4968696 27192671

[63] Lahav M Uziel O Kestenbaum M Fraser A Shapiro H Radnay J Nonmyeloablative conditioning does not prevent telomere shortening after allogeneic stem cell transplantation. Transplantation (2005) 80(7):969–76. 10.1097/01.TP.0000173649.99261.DF 16249747

[64] Armenian SH Sun CL Kawashima T Arora M Leisenring W Sklar CA Long-term health-related outcomes in survivors of childhood cancer treated with HSCT versus conventional therapy: a report from the Bone Marrow Transplant Survivor Study (BMTSS) and Childhood Cancer Survivor Study (CCSS). Blood (2011) 118(5):1413–20. 10.1182/blood-2011-01-331835 PMC3152502 21652685

[65] Chovanec M Cierna Z Miskovska V Machalekova K Kalavska K Rejlekova K Systemic immune-inflammation index in germ-cell tumours. Br J Cancer (2018) 118(6):831–8. 10.1038/bjc.2017.460 PMC5877428 29485980

[66] Rossen PB Pedersen AF Zachariae R Von Der Maase H Health-related quality of life in long-term survivors of testicular cancer. J Clin Oncol (2009) 27:5993–9. 10.1200/JCO.2008.19.6931 19858403

[67] Oechsle K Hartmann M Mehnert A Oing C Bokemeyer C Vehling S Symptom burden in long-term germ cell tumour survivors. Support Care Cancer (2016) 24:2243–50. 10.1007/s00520-015-3026-9 26576967

[68] Sprauten M Haugnes HS Brydøy M Kiserud C Tandstad T Bjøro T Chronic fatigue in 812 testicular cancer survivors during longterm follow-up: increasing prevalence and risk factors. Ann Oncol (2015) 26:2133–40. 10.1093/annonc/mdv328 26265167

[69] Fung C Fossa SD Williams A Travis LB Long-term morbidity of testicular cancer treatment. Urol Clin North Am Elsevier Inc (2015) 42:393–408. 10.1016/j.ucl.2015.05.002 26216826

[70] Vehling S Mehnert A Hartmann M Oing C Bokemeyer C Oechsle K Anxiety and depression in long-term testicular germ cell tumor survivors. Gen Hosp Psychiatry (2016) 38:21–5. 10.1016/j.genhosppsych.2015.09.001 26439320

[71] Smith AB Rutherford C Butow P Olver I Luckett T Grimison P A systematic review of quantitative observational studies investigating psychological distress in testicular cancer survivors. Psycho-Oncology (2018) 27:1129–37. 10.1002/pon.4596 29171109

[72] Fleer J Quality of Life of Testicular Cancer Survivors. Groningen (2006). 168 p. [s.n.].

[73] Smith AB Butow P Olver I Luckett T Grimison P Toner GC The prevalence, severity, and correlates of psychological distress and impaired health-related quality of life following treatment for testicular cancer: a survivorship study. J Cancer Surviv (2016) 10(2):223–33. 10.1007/s11764-015-0468-5 26178326

[74] Kreiberg M Bandak M Lauritsen J Andersen KK Skøtt JW Johansen C Psychological stress in long-term testicular cancer survivors: a Danish nationwide cohort study. J Cancer Surv (2020) 14(1):72–9. 10.1007/s11764-019-00835-0 31748852

[75] Dahl AA Østby-Deglum M Oldenburg J Bremnes R Dahl O Klepp O Aspects of posttraumatic stress disorder in long-term testicular cancer survivors: cross-sectional and longitudinal findings. J Cancer Surviv (2016) 10(5):842–9. 10.1007/s11764-016-0529-4 26920871

[76] Glaser R Kiecolt-Glaser JK Stress-induced immune dysfunction: Implications for health. Nat Rev Immunol (2005) 5(3):243–51. 10.1038/nri1571 15738954

[77] Bauer ME Chronic stress and immunosenescence: a review. Neuroimmunomodulation (2008) 15:241–50. 10.1159/000156467 19047801

[78] Cohen S Tyrrell DA Smith AP Psychological stress and susceptibility to the common cold. N Engl J Med (1991) 325:606–12. 10.1056/NEJM199108293250903 1713648

[79] Reed R Stress and immunological aging. Curr Opin Behav Sci (2019) 28:38–43. 10.1016/j.cobeha.2019.01.012 31179376 PMC6548512

[80] Epel ES Crosswell SD Mayer SE Prather AA Slavich GM Puterman E More than a feeling: a unified view of stress measurement for population science. Front Neuroendocrinol (2018) 49:146–69. 10.1016/j.yfrne.2018.03.001 PMC6345505 29551356

[81] Bosch JA Fischer JE Fischer JC Psychologically adverse work conditions are associated with CD8+ T cell differentiation indicative of immunesenescence. Brain Behav Immun (2009) 23:527–34. 10.1016/j.bbi.2009.02.002 19217939

[82] Gouin JP Hantsoo L Kiecolt-Glaser JK Immune dysregulation and chronic stress among older adults. Neuroimmunomodulation (2008) 5:251–9. 10.1159/000156468 PMC2676338 19047802

[83] de Punder K Heim C Wadhwa PD Entringer S Stress and immunosenescence: The role of telomerase. Psychoneuroendocrinology (2019) 101:87–100. 10.1016/j.psyneuen.2018.10.019 30445409 PMC6458519

[84] Bauer ME Stress, glucocorticoids and ageing of the immune system. Stress (2005) 8(1):69–83. 10.1080/10253890500100240 16019599

[85] Garcia-Bueno B Caso JR Leza JC Stress as a neuroinflammatory condition in brain: damaging and protective mechanisms. Neurosci Biobehav Rev (2008) 32(6):1136–51. 10.1016/j.neubiorev.2008.04.001 18468686

[86] Maes M Yirmyia R Noraberg J Brene S Hibbeln J Perini G The inflammatory & neurodegenerative (I&ND) hypothesis of depression: leads for future research and new drug developments in depression. Metab Brain Dis (2009) 24(1):27–53. 10.1007/s11011-008-9118-1 19085093

[87] Kubera M Obuchowicz E Goehler L Brzeszcz J Maes M In animal models, psychosocial stress-induced (neuro)inflammation, apoptosis and reduced neurogenesis are associated to the onset of depression. Prog Neuropsychopharmacol Biol Psychiatry (2011) 35:744–59. 10.1016/j.pnpbp.2010.08.026 20828592

[88] Graham JE Christian LM Kiecolt-Glaser JK Stress, age, and immune function: toward a lifespan approach. J Behav Med (2006) 29(4):389–400. 10.1007/s10865-006-9057-4 16715331 PMC2805089

[89] Epel ES Psychological and metabolic stress: a recipe for accelerated cellular aging? Hormones (2009) 8(1):7–22. 10.14310/horm.2002.1217 19269917

[90] Engeland CG Salivary biomarkers in psychoneuroimmunology. Curr Opin Behav Sci (2019) 28:58–65. 10.1016/j.cobeha.2019.01.007 32215283 PMC7094032

[91] Flory JD Yehuda R Comorbidity between post-traumatic stress disorder and major depressive disorder: alternative explanations and treatment considerations. Dialogues Clin Neurosci (2015) 17:141–50. 10.31887/DCNS.2015.17.2/jflory PMC4518698 26246789

[92] Baker DG Nievergelt CM O’Connor DT Biomarkers of PTSD: neuropeptides and immune signaling. Neuropharmacology (2012) 62:663–73. 10.1016/j.neuropharm.2011.02.027 21392516

[93] Seiler A Fagundes CP Christian LM “The Impact of Everyday Stressors on the Immune System and Health”. In: Choukèr A, editor. Stress Challenges and Immunity in Space. Cham: Springer (2020). 10.1007/978-3-030-16996-1_6

[94] Lohr JB Palmer BW Eidt CA Aailaboyina S Mausbach BT Wolkowitz OM Is post-traumatic stress disorder associated with premature senescence? A review of the literature. Am J Geriatr Psychiatry (2015) 23:709–25. 10.1016/j.jagp.2015.04.001 PMC4568841 25959921

[95] Avetyan D Zakharyan R Petrek M Arakelyan A Telomere shortening in blood leukocytes of patients with posttraumatic stress disorder. J Psychiatr Res (2019) 111:83–8. 10.1016/j.jpsychires.2019.01.018 30685566

[96] Aiello AE Dowd JB Jayabalasingham B Feinstein L Uddin M Simanek AM PTSD is associated with an increase in aged T cell phenotypes in adults living in Detroit. Psychoneuroendocrinology (2016) 67:133–41. 10.1016/j.psyneuen.2016.01.024 PMC4826331 26894484

[97] Aiello AE Feinstein L Dowd JB Pawelec G Derhovanessian E Galea S Income and markers of immunological cellular aging. Psychosomatic Med (2016) 78:657–66. 10.1097/PSY.0000000000000320 PMC4927391 27187853

[98] Prather AA Epel ES Portela Parra E Coccia M Puterman E Aiello AE Associations between chronic caregiving stress and T cell markers implicated in immunosenescence. Brain Behav Immun (2018) 73:546–9. 10.1016/j.bbi.2018.06.019 PMC6129414 29935942

[99] Antoni MH Psychosocial intervention effects on adaptation, disease course and biobehavioral processes in cancer. Brain Behav Immun (2013) 30 Suppl:S88–98. 10.1016/j.bbi.2012.05.009 PMC3444659 22627072

[100] Lubberts S Meijer C Demaria M Gietema JA Early ageing after cytotoxic treatment for testicular cancer and cellular senescence: Time to act. Crit Rev Oncol Hematol (2020) 151:102963. 10.1016/j.critrevonc.2020.102963 32446180

[101] Gietema JA Meinardi MT Messerschmidt J Gelevert T Alt F Uges DR Circulating plasma platinum more than 10 years after cisplatin treatment for testicular cancer. Lancet (2000) 355(9209):1075–6. 10.1016/S0140-6736(00)02044-4 10744098

[102] Boer H Proost JH Nuver J Bunskoek S Gietema JQ Geubels BM Long-term exposure to circulating platinum is associated with late effects of treatment in testicular cancer survivors. Ann Oncol (2015) 26(11):2305–10. 10.1093/annonc/mdv369 PMC4621032 26347114

[103] Sprauten M Darrah TH Peterson DR Campbell ME Hannigan RE Cvancarova M Impact of long-term serum platinum concentrations on neuro- and ototoxicity in Cisplatin-treated survivors of testicular cancer. J Clin Oncol (2012) 30(3):300–7. 10.1200/JCO.2011.37.4025 PMC3269954 22184390

[104] Schepisi G De Padova S De Lisi D Casadei C Meggiolaro E Ruffilli F Psychosocial Issues in Long-Term Survivors of Testicular Cancer. Front Endocrinol (2019) 10:113. 10.3389/fendo.2019.00113 PMC6397854 30858829

[105] Rowland DL Incrocci L Handbook of Sexual and Gender Identity Disorders. Hoboken, NJ: John Wiley & Sons (2008) p. 68–99. 10.1002/9781118269978

[106] Dahl AA Bremnes R Dahl O Klepp O Wist E Fosså SD Is the sexual function compromised in long-term testicular cancer survivors? Eur Urol (2007) 52:1438–47. 10.1016/j.eururo.2007.02.046 17350159

[107] Gunnes MW Lie RT Bjørge T Ghaderi S Ruud E Syse A Reproduction and marriage among male survivors of cancer in childhood, adolescence and young adulthood: a national cohort study. Br J Cancer (2016) 114:348–56. 10.1038/bjc.2015.455 PMC4742584 26794280

[108] Bandak M Lauritsen J Johansen C Kreiberg M Skøtt JW Agerbaek M Sexual Function in a Nationwide Cohort of 2,260 Survivors of Testicular Cancer after 17 Years of Followup. J Urol (2018) 200(4):794–800. 10.1016/j.juro.2018.04.077 29730199

[109] Bandak M Jørgensen N Juul A Vogelius IR Lauritsen J Kier MG Testosterone deficiency in testicular cancer survivors - a systematic review and meta-analysis. Andrology (2016) 4(3):382–8. 10.1111/andr.12177 27009402

[110] Sprauten M Brydøy M Haugnes HS Cvancarova M Bjøro T Bjerner J Longitudinal serum testosterone, luteinizing hormone, and follicle-stimulating hormone levels in a population-based sample of long-term testicular cancer survivors. J Clin Oncol (2014) 32(6):571–8. 10.1200/JCO.2013.51.2715 24419125

[111] Haugnes HS Wethal T Aass N Dahl O Klepp O Langberg CW Cardiovascular risk factors and morbidity in long-term survivors of testicular cancer: a 20-year follow-up study. J Clin Oncol (2010) 28:4649–57. 10.1200/JCO.2010.29.9362 20855830

[112] La Vignera S Cannarella R Duca Y Barbagallo F Burgio G Compagnone M Hypogonadism and Sexual Dysfunction in Testicular Tumor Survivors: A Systematic Review. Front Endocrinol (Lausanne) (2019) 10:264. 10.3389/fendo.2019.00393 31133982 PMC6513875

[113] Bandak M Jørgensen N Juul A Lauritsen J Kreiberg M Oturai PS A randomized double-blind study of testosterone replacement therapy or placebo in testicular cancer survivors with mild Leydig cell insufficiency (Einstein-intervention). BMC Cancer (2017) 17(1):461. 10.1186/s12885-017-3456-5 28673265 PMC5494856

[114] Skøtt JW Lauritsen J Kreiberg M Daugaard G Bandak M Quality of Life in Long-Term Testicular Cancer Survivors With Compensated Leydig Cell Dysfunction. Clin Genitourin Cancer (2019) 17(1):e65–71. 10.1016/j.clgc.2018.09.004 30293923

[115] Vigen R O'Donnell CI Barón AE Grunwald GK Maddox TM Bradley SM Association of testosterone therapy with mortality, myocardial infarction, and stroke in men with low testosterone levels. JAMA (2013) 310(17):1829–36. 10.1001/jama.2013.280386 24193080

[116] Morgentaler A Miner MM Caliber M Guay AT Khera M Traish M Testosterone therapy and cardiovascular risk: advances and controversies. Mayo Clin Proc (2015) 90(2):224–51. 10.1016/j.mayocp.2014.10.011 25636998

[117] Tsai EC Matsumoto AM Fujimoto WY Boyko EJ Association of Bioavailable, Free, and Total Testosterone With Insulin Resistance Influence of sex hormone-binding globulin and body fat. Diabetes Care (2004) 27(4):861–8. 10.2337/diacare.27.4.861 15047639

[118] Liao CH Lin FY Wu YN Chiang HS Androgens inhibit tumor necrosis factor-α-induced cell adhesion and promote tube formation of human coronary artery endothelial cells. Steroids (2012) 77:7:756–64. 10.1016/j.steroids.2012.03.014 22504554

[119] Kalinchenko SY Tishova YA Mskhalaya GJ Gooren LJ Giltay EJ Saad F Effects of testosterone supplementation on markers of the metabolic syndrome and inflammation in hypogonadal men with the metabolic syndrome: the double-blinded placebo-controlled Moscow study. Clin Endocrinol (Oxf) (2010) 73(5):602–12. 10.1111/j.1365-2265.2010.03845.x 20718771

[120] Chrysohoou C Panagiotakos D Pitsavos C Siasos G Oikonomou E Varlas J Low Total Testosterone Levels are Associated With the Metabolic Syndrome in Elderly Men: The Role of Body Weight, Lipids, Insulin Resistance, and Inflammation; The Ikaria Study. Rev Diabetes Stud (2013) 10(1):27–38. 10.1900/RDS.2013.10.27 PMC3932069 24172696

[121] Maggio M Basaria S Ble A Lauretani F Bandinelli S Ceda GP Correlation between testosterone and the inflammatory marker soluble interleukin-6 receptor in older men. J Clin Endocrinol Metab (2006) 91(1):345–7. 10.1210/jc.2005-1097 16263825

[122] Horwich A Fossa SD Huddart R Dearnaley DP Stenning S Aresu M Second cancer risk and mortality in men treated with radiotherapy for stage I seminoma. Br J Cancer (2014) 110:256–63. 10.1038/bjc.2013.551 PMC3887279 24263066

[123] van den Belt-Dusebout AW de Wit R Gietema JA Horenblas S Louwman M Ribot JG Treatment-specific risks of second malignancies and cardiovascular disease in 5-year survivors of testicular cancer. J Clin Oncol (2007) 25:4370–8. 10.1200/JCO.2006.10.5296 17906202

[124] Fung C Fossa SD Milano MT Oldenburg J Travis LB Solid tumors after chemotherapy or surgery for testicular nonseminoma: a population-based study. J Clin Oncol (2013) 31:3807–14. 10.1200/JCO.2013.50.3409 PMC3795890 24043737

[125] Howard R Gilbert E Lynch CF Hall P Storm H Holowaty E Risk of leukemia among survivors of testicular cancer: a population-based study of 42,722 patients. Ann Epidemiol (2008) 18:416–21. 10.1016/j.annepidem.2008.01.003 PMC4034178 18433667

